# Interpretable and parameter optimized ensemble model for knee osteoarthritis assessment using radiographs

**DOI:** 10.1038/s41598-021-93851-z

**Published:** 2021-07-12

**Authors:** Mohammed Bany Muhammad, Mohammed Yeasin

**Affiliations:** Department of Electrical and Computer Engineering, Herff College, University of Memphis, Memphis, TN USA

**Keywords:** Computational models, Image processing, Machine learning

## Abstract

Knee osteoarthritis (KOA) is an orthopedic disorder with a substantial impact on mobility and quality of life. An accurate assessment of the KOA levels is imperative in prioritizing meaningful patient care. Quantifying osteoarthritis features such as osteophytes and joint space narrowing (JSN) from low-resolution images (i.e., X-ray images) are mostly subjective. We implement an objective assessment and quantification of KOA to aid practitioners. In particular, we developed an interpretable ensemble of convolutional neural network (CNN) models consisting of three modules. First, we developed a scale-invariant and aspect ratio preserving model to localize Knee joints. Second, we created multiple instances of "hyperparameter optimized" CNN models with diversity and build an ensemble scoring system to assess the severity of KOA according to the Kellgren–Lawrence grading (KL) scale. Third, we provided visual explanations of the predictions by the ensemble model. We tested our models using a collection of 37,996 Knee joints from the Osteoarthritis Initiative (OAI) dataset. Our results show a superior (13–27%) performance improvement compared to the state-of-the-art methods.

## Introduction

Osteoarthritis (OA) is a common form of degenerative joint disorder characterized by functional impairment and chronic pain. OA has a profound impact on the economy, individuals, and society. The economic direct and indirect burden in the US is estimated at around $100 billion^1^. OA affects 12.1% (32.5 million) of the adult population and is considered the 5th cause of disability in the US^1,2^. It ranked in 2013 as the second most costly condition to treat in US hospitals after Septicemia^3^. The social burden of OA is represented by the increasing functional impairments and loss of independence in older communities^2^.

KOA is the most prevalent type of OA and represents the 11th cause leading to disability^1,4^. The degenerative nature of KOA imposes increasing costs upon patients ($4000–$5000 for moderate levels of KOA to $8000–$10,000 for severe cases of KOA)^5^. KOA is more prevalent among two categories, the first, females aged 60 years or more when compared to males of the same age^6^. The second category includes all younger people characterized by obesity^7^.

In general, diagnosed cases with KOA shows a marginally positive association with age and weight.

Physicians rely on radiographs and patient clinical records to assess the KOA severity level. Radiographic images are the primary means to determine the class of osteoarthritis and will be used for a long time due to many reasons that include affordability and availability. The low-resolution radiographs limit the physicians' ability to quantify the joint OA abnormalities: JSN, Sclerosis, Cyst, Attrition, Chondrocalcinosis, and Osteophytes.

**Figure 1:**
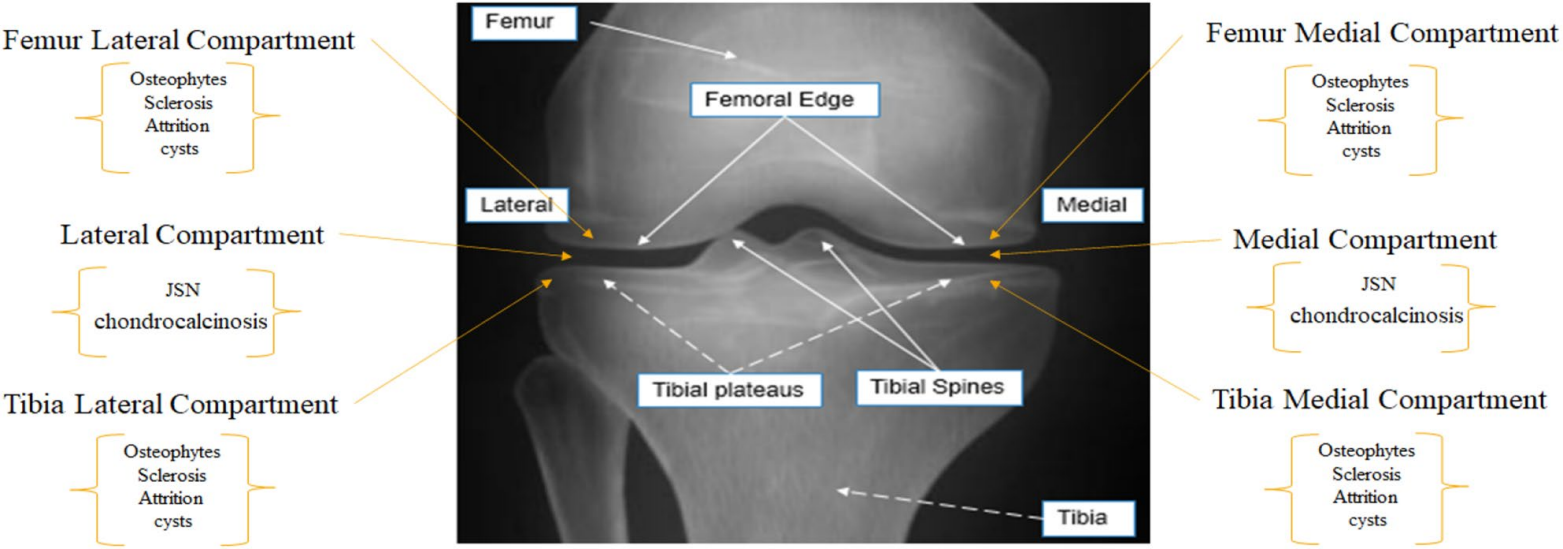
Radiographic knee anatomy and OA feature locations.

In KOA, JSN, Chondrocalcinosis, and Attrition are quantified at the lateral compartment and medial compartment. JSN and attrition are graded based on Osteoarthritis Research Society International (OARSI) with a four-level grading system^8^. In contrast, chondrocalcinosis is graded based on OARSI with a two-level grading system. All other OA features are detected/quantified at four points: the lateral femur compartment, medial femur compartment, tibia lateral compartment, and tibia medial compartment. Osteophytes and Sclerosis are graded based on OARSI with a four-level grading system. Meanwhile, the cyst is graded based on OARSI with a two-level grading system (Fig. 1) for feature location.

**Figure 2:**
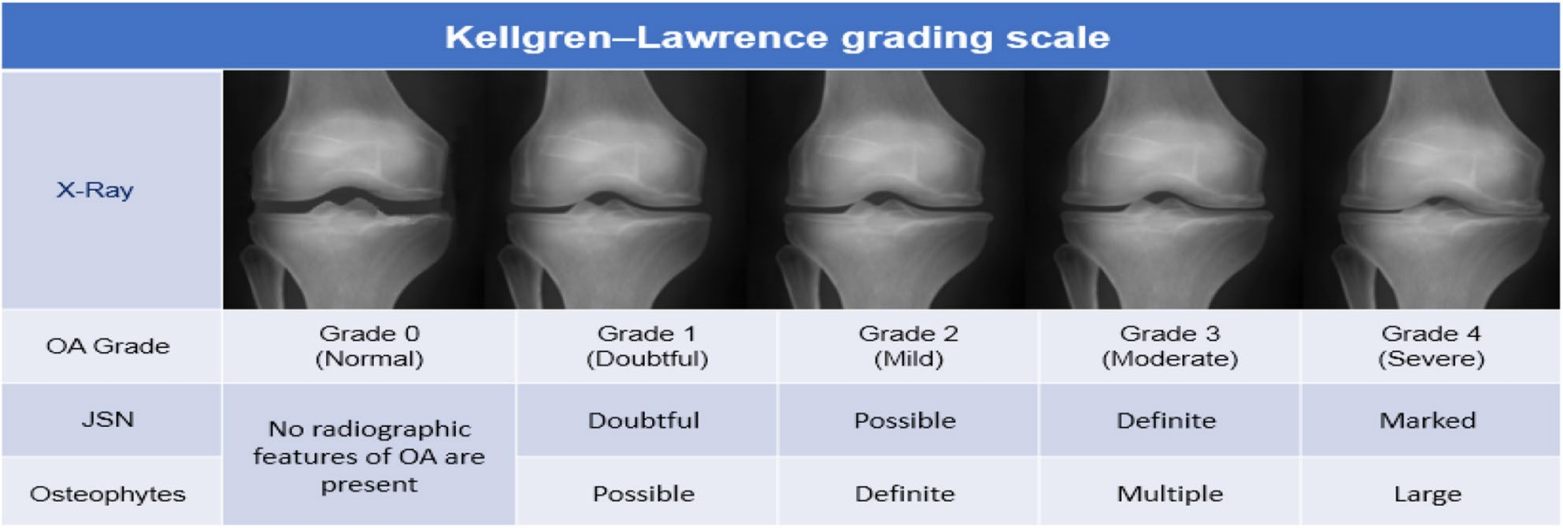
KL starts with KL = 0 representing normal and KL = 4 representing most severe cases of OA.

In the process of scoring KOA, Physicians map quantified features from X-ray images (Osteophytes and JSN) into a 5 level grading system based on the KL grading scale^9^. ‘Grade 0' corresponds to the normal knee, 'Grade 1' corresponds to doubtful KOA, 'Grade 2' corresponds to mild KOA, 'Grade 3' corresponds to moderate KOA, and 'Grade 4' corresponds to severe case of KOA (Fig. 2).

Since the 1950s, researchers have used different methods to quantify the KOA severity from radiographs. In general, we can group most of the semi-automated methods into three categories. The geometric approaches utilize variants of image processing techniques such as edge and contour detectors, vertical intensity profiling, and spectral clustering to characterize the JSN^10–12^. Such methods failed to account for most OA features such as Sclerosis, cyst, attrition, chondrocalcinosis, and osteophytes. That motivated researchers to turn to classical machine learning and pattern recognition algorithms for KOA severity assessment to improve the assessment accuracy. They utilized methods such as (but are not limited to): Self-organizing Map (SOM)^13^, Weighted neighbor distance using the compound hierarchy of algorithms representing morphology (WND-CHARM)^14^ using features like Gray Level Co-occurrence Matrix (GLCM), histogram of oriented gradients (HOG), Multi-scale Histograms, Zernike moment, First Four Moments, Tamura Texture, and Chebyshev Statistics. In general, this approach failed to match the performance achieved by radiographs expert readers.

Since the 2010s, with the rise of deep learning (DL) in various computer vision tasks, the third line of work based on CNN modeling dominates the research field in the scope of such problems. Antony et al. introduced the first CNN-based architecture for scoring KOA severity based on radiographs^15,16^. Using Antony's two-step procedure (joint localization and OA severity quantification), several methods utilized different CNN-based architectures to quantify KOA based on radiographs. CNN-based methods included deep Siamese CNN^15^, deep ensemble CNN^17^, graph CNN^18^, attention-based end-to-end CNN architectures^19^, improved Faster R-CNN^20^, and densely connected CNN^21–23^. In general, all methods implementing CNN for KOA severity assessment outperformed all previous methods and achieved performance comparable to human (quadratic Kappa coefficient (0.66^24^, 0.66^25^, 0.67^26^) calculated for expert radiograph readers for OA severity assessment), and that reflects the difficulty for both human and CNN based models in quantifying the severity level of KOA.

To the best of our knowledge, this is the first study to design and implement a reliable, interpretable, parameter optimized, and fully automated CNN-based model for KOA severity assessment based on radiographs from all six clinical visits in the OAI. Our systematic approach implements: (i) data preprocessing to enhance radiographs, (ii) data augmentation to increase the number of images used for training purposes, (iii) modify single shot multi-box detector (SSD)^27^ to localize knee joints with high accuracy, (iv) address class imbalance to obtain optimal bias-variance trade-off, (v) assess the severity of KOA using stacked CNN-based ensemble model, and locate OA features using Eigen-CAM^28,29^. The proposed approach is not disease-specific and is expected to be equally suitable for modeling any other disease if similar care is taken in data processing and building parameter-optimized interpretable models.

## Results

### Data processing

OAI dataset contains radiographs collected at six clinical visits. The baseline visit contains 4796 radiographs for 4796 patients. The number of collected radiographs decreases with each clinical visit due to patients dropping from the OAI study. The total number of collected radiographs in six clinical visits is 22,279. Since each knee is quantified separately, we have a total of 44,558 different knees. To train the modified SSD model, we have manually annotated (locate the coordinates of an abounding box that enclose the kneecap area) 600 images with a total of 1200 kneecaps.

### Results of knee joint detection

We split annotated radiographs equally for training, validating, and testing models. We used the train and validation set to fine-tune the MobileNet V1 in the SSD architecture and used the test set to quantify the localization results based on the Jaccard index [defined as the area of intersection over the area of union (IOU)].

In General, the modified SSD architecture detects 43,981 joints and fails to detect 577 knee joints. The modified SSD architecture is an exceptionally accurate 95% IOU based on the 0.75 Jaccard index. Table 1 shows a comparison with state-of-the-art methods for the task of knee joint localization. In general, the modified SDD model show (2–4%) performance improvement when compared to state-of-the-art DL-based methods (last 2 years) for the task of knee localization, and most importantly, it maximizes the use of available radiographs for the following process.

**Table 1:** Comparison of the knee localization task.

Year	Method	IOU
2016	Reference^15^	0.386
2017	Reference^16^	0.830
2018	Reference^4^	0.830
2019	Reference^17^	0.910
2019	Reference^20^	0.924
2020	Reference^23^	0.930
2020	Ours	0.950

### Performance of diverse CNN base models

After knee joint localization, we excluded all radiographs that underwent total knee replacement and excluded radiographs with no KL level. The total number of localized knee joints available is 37,996. To train, validate, and test models, we used a 60%–20%–20% split ratio for training, validation, and testing, respectively. We used the training and validation set to train (22,796 radiographs) and validate (7601 radiographs) the base models. And the test set to evaluate the stacked ensemble learner. Table 2 shows the performance of CNN base models ranging from 0.69 to 0.71% average class accuracy. Table 4 reports performances of state-of-the-art methods. Any base model's performance outperforms most of the reported work for the KOA severity assessment (Table 4) irrespective of simplified design and shallower design for some base models. This performance improvement is attributed to training using a more significant number of radiographs and building parameter-optimized models.

**Table 2:** Classification metrics for the base models.

Base model	Average precision	Average recall	F1 score	Average class accuracy
Base Model 1	0.69	0.68	0.68	0.69
Base Model 2	0.70	0.68	0.68	0.69
Base Model 3	0.69	0.69	0.68	0.69
Base Model 4	0.72	0.70	0.71	0.71
Base Model 5	0.70	0.69	0.69	0.69
Base Model 6	0.72	0.70	0.71	0.71

### Results of ensemble of CNN models

Ensemble models rely on the diversity of base models to enhance performance. The diversity of 1 represents complete disagreement between base models, and 0 represents complete agreement. With 0 diversity between base models (all base models have the same performance), the ensemble learners' accuracy is minimum. Theoretically higher the diversity of base models learned patterns higher the performance of ensemble learners^30^. To measure the diversity between base models, we used the modified non-pairwise measure interrater reliability (κ) to account for the chance in agreement. The overall measured diversity for base models is 0.3359.

To train the stacked ensemble model (Fig. 7), we used all CNN base models' output as inputs to the super learner. We experimented with support vector machines (SVM)^31^, Random Forest (RF)^32^, and Gradient Boosting Machines (GBM)^33^ for decision level fusion to create the ensemble model. SVM provides a slightly better average class accuracy (1%) compared to other methods. To validate the SVM classifier as a super learner, we used the test radiographs (7599 radiographs).

To tune the SVM classifier, we set the categorical range of the kernel to ('linear', 'poly', 'rbf', 'sigmoid'), the range of the regularization parameter (C) between (0–10), and the range of kernel coefficient gamma) between (0–1). The optimal parameters that maximize the accuracy are C = 1, Kernel = 'rbf’ and Gamma = 0.02.

**Figure 3:**
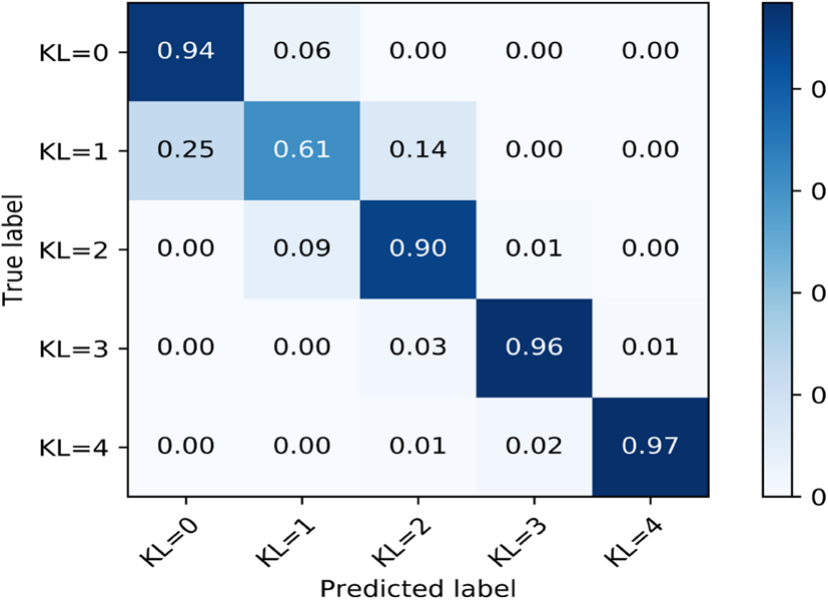
Normalized confusion matrix for the super learner model.

In Table 3 and Fig. 3, we present the performance and the normalized confusion matrix, respectively. Measured sensitivity for normal, doubtful, mild, moderate, and severe OA is 94%, 61%, 90%, 96%, and 97%, respectively. The model had a weighted average F1 score of 87%.

**Table 3:** Classification metrics for the super learner.

KL level	Precision	Recall	F1 Score	Support
KL = 0	0.89	0.94	0.90	3041
KL = 1	0.72	0.61	0.66	1457
KL = 2	0.88	0.90	0.89	1825
KL = 3	0.97	0.96	0.96	986
KL = 4	0.96	0.97	0.96	290
Accuracy			0.87	7599
Macro Avg	0.88	0.88	0.88	7599
Weighted Avg	0.86	0.87	0.87	7599

The normalized confusion matrix tested on 7599 radiographs shows near-perfect diagonal matrix except for the doubtful level [KL = 1]. It also shows zero confusion among non-adjacent levels of OA, and that reflects the superior generalization of the model.

Using the super learner in stacking ensemble learning boosted the average class accuracy to 0.87, representing a 7% improvement over a simple majority vote ensemble learning using the same base models.

To demonstrate the proposed pipeline's effectiveness, we presented in Table 4 results of the stacked CNN ensemble model compared with the state-of-the-art methods trained and validated on standard OA datasets.

**Table 4:** Comparison with state-of-the-art DL-based methods for KOA severity assessment task.

Year	Method	Validation set size	Average class accuracy (%)
2016	Reference^15^	2686	59.60
2017	Reference^16^	4400	62.29
2018	Reference^4^	2957	66.70
2019	Reference^17^	1890	69.50
2019	Reference^19^	1495	64.3
2019	Reference^20^	1385	74.3
2020	Reference^23^	1770	60.0
2020	Reference^22^	4090	71.0
2020	Ours	7599	87.0

All methods listed in Table 4 except for Ref.^17^ implement different variations of typical CNN architectures trained to predict the severity of KOA, Ref.^17^ utilize a simple majority vote for a number of shallow CNN architectures to predict the severity of KOA meanwhile our work utilizes ensemble learning from optimized CNN models and employ the SVM as a super learner to minimize the confusion at higher levels of KOA severity levels.

In summary, we achieve a 95% average IOU in localizing knee joint and 87.0% average class accuracy for the KOA severity assessment using the entire set of radiographs in the OAI dataset for all six clinical visits.

### Prediction visual explanations

Tools that aim at providing visual explanations of CNN prediction can play an essential role in the design process by verifying generalization capability. Also, it can provide a measure of trustworthiness in the CNN-Based models for the end-users.

**Figure 4:**
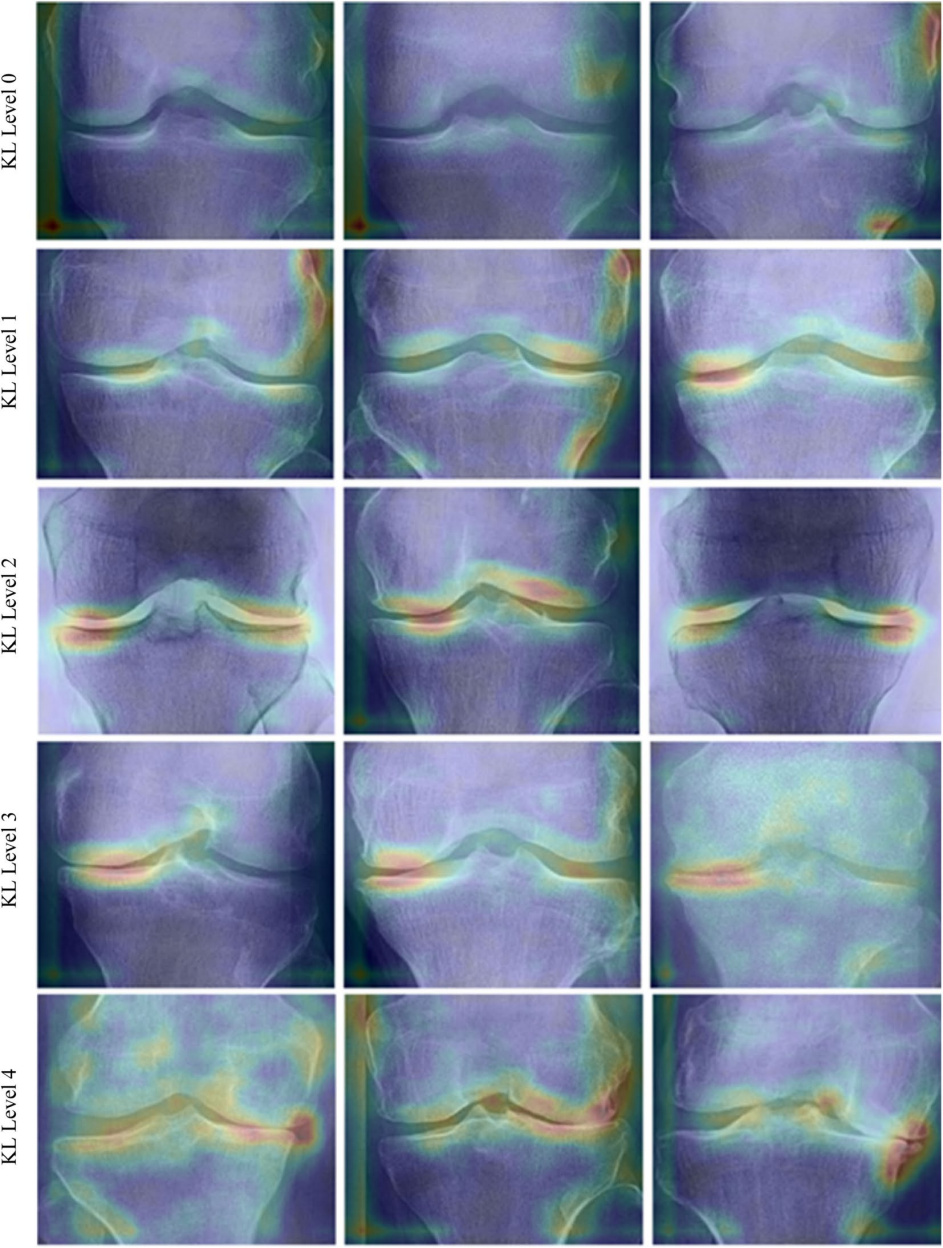
Ensemble CNN prediction explanations produced using Eigen-CAM for three sample images for different severity levels.

To explore learned patterns from the Stacked ensemble CNN model, we used Eigen-CAM to generate visual explanations of the ensemble CNN for different grades of KL, as shown in (Fig. 4). We can identify consistent patterns for each class level and different patterns across different class levels of KOA. Such visualization provides qualitative evidence of learning (no overfitting) and helps identify relevant features that match expected OA features (JSN and Osteophytes) in the joint medial and lateral margins.

## Discussion

This work proposed an end-to-end optimized and interpretable DL-based CNN architecture that fully automates the process of KOA severity assessment using models built using the OAI dataset and tested on 7599 knee joints that the models never saw. First, the proposed model achieves an average class accuracy of 87%. Second, a 21% improvement over the average weighted quadratic kappa factor compared with expert readers in the KOA severity assessment. These results indicate the superiority of the model over expert performance. Third, 0.87% F1 Score and that by far exceeds any reported results, we present the robust metrics F1 Score due to imbalance in class levels in the OAI dataset and its ability to reflect both Recall (how good is the learned patterns) and Precision (better accuracy).

Building deep CNN models requires massive data volume. Arguably the deeper the model better the performance and hence the better the generalization capability. In general, the number of learnable parameters is proportional to the CNN model's depth, and hence more data is required to learn model parameters. In this work, we build deeper models compared to our previous work^17^ by utilizing all radiographs from the longitudinal OAI dataset. We also implement data augmentation methods that do not affect OA features and provide adequate variation to generate virtual radiographs for training purposes only.

The process of building reliable CNN-based models requires, first, building a diverse set of models that exceeds the quadratic kappa factor among human experts judging the severity of KOA. We achieved that by utilizing all best practices such as data preprocessing, Bayesian hyper-parameter optimization to optimize and fine-tune all parameters, and multi-level learning such as stacking. Second, the process requires robustness, and that is obtained by augmenting the training data and paying attention to the Bias-Variance trade-off. Third, rigorous performance evaluation of models by examining the test accuracy, precision, and recall (the ability of not picking garbage), consistency of prediction using F1 score, and generalization capability using AUC (higher AUC → better generalization). Fourth, it requires the ability to decode error, and finally, the process requires interpretability (visual explanations of prediction), which is achieved using Eigen-CAM tools.

Even though we presented the best performance among reported work, we have noted three limitations to our work. First, our models provided a lower performance in classifying KL = 1 compared to other KL levels. The same observation can be noted in all other reported methods. This consistent lower performance is justified as the KL grading scale is flawed and relies on JSN and Osteophytes only and discards OA features such as Sclerosis, Cyst, Attrition, and Chondrocalcinosis. Second, we did train and validate our models based on the OAI dataset only since we could not obtain datasets like MOST or KNHANES, and we firmly believe that obtaining radiographs from other datasets will enable us to achieve higher performance and helps show a better generalization. Third, a final limitation to be noted is that this work entirely relies on radiographs and does not synthesize other modalities, such as patient clinical records.

In general, we presented a reliable and fully automated pipeline that can function as a decision support system in this work. This work will assist experts in achieving more objective and less subjective in assessing KOA. Also, the visualization of model prediction using Eigen-CAM will build trust in determining the KOA severity level.

## Methods

**Figure 5:**
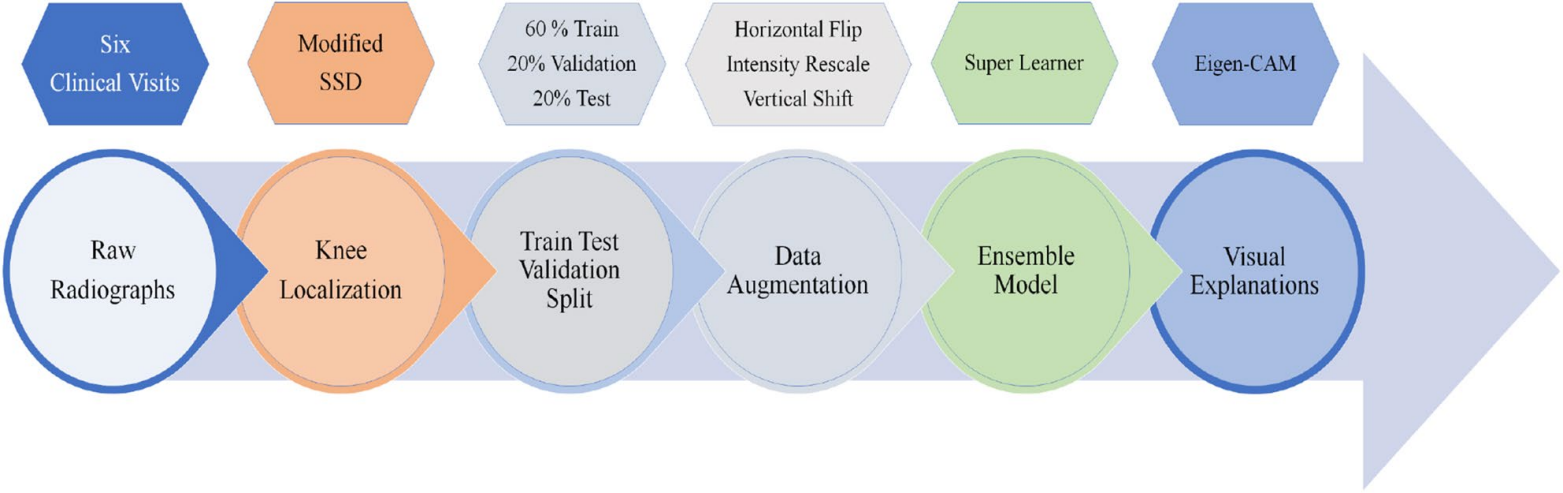
Proposed pipeline for workflow. (**a**) Data concatenation for baseline visit and follow-up visits, (**b**) Kneecap area localization, (**c**) Train test validation split (**d**) Data augmentation, (**e**) Ensemble learning, (**f**) Visual explanation of predicted classes.

In (Fig. 5), we show the computational building blocks used for modeling of OA severity assessment based on radiographs from the OAI dataset. The proposed pipeline in (Fig. 5) is a sequential step by step, optimized for maximum performance. First, we extract and process all radiographs from six clinical visits. Second, we localize knee joints. Third, we split the data between training, validation, and test to train and evaluate multi-level architecture. Fourth, we implement data augmenting for the training dataset. Fifth, we used hyperparameter optimization at every level of the training and evaluation of the stacked ensemble model. Finally, we visualize learned patterns from radiographs.

### Data

The Osteoarthritis Initiative (OAI) is a multi-center, longitudinal, prospective observational study collected by the University of California San Francisco (UCSF), sponsored by the National Institute of Health (NIH), and labeled (OA severity level) by Boston University X-ray reading center (BU). The data set aims at a better understanding of how to prevent and treat KOA. The OAI dataset contains data for 4796 individuals (41.5%) men and (58.5%) women aged between 45–79 years old^34^. The dataset is open for public access at http://www.oai.ucsf.edu/. The Radiographs used in this study from the OAI dataset include baseline and five clinical visits. Radiographs in OAI are bilateral Posterior-Anterior (PA) fixed flexion knee X-ray images provided in a digital imaging and communications in medicine (DICOM) format. Table 5 presents the KL distribution, the number of available radiographs, and the percentage of the missing KL levels in baseline and the follow-up clinical visits.

**Table 5:** Distribution of KOA severity levels in the OAI dataset.

Visiting cycle	KL0	KL1	KL2	KL3	KL4	Missing KL	Available Radiographs
Baseline	3448	1597	2374	1239	295	639 (6.66%)	4796
12-month	3113	1445	2221	1230	355	1228 (12.8%)	3660
36-month	2735	1252	1986	1147	377	2095 (21.84%)	4106
48-month	2606	1196	1884	1062	389	2455 (25.59%)	3700
72-month	1866	1007	471	201	26	6021 (62.77%)	3085
96-month	1899	987	488	239	47	5932 (61.84%)	2932

### Data preprocessing

The radiographs in the OAI dataset are in a DICOM format. We convert all images into the Joint Photographic Experts Group (JPEG) format for subsequent processing convenience. Then we equalize intensity in each image using contrast limited adaptive histogram equalization (CLAHE) with a tile grid size of (8 × 8) and a clipping limit of 2. CLAHE improves contrast in images by redistributing the lightness values in the image and also enhances edges in local regions. In the last step, we scale all image intensities to (0–1) range.

The total number of Knee X-ray images available in the entire OAI data set is 22,279. The radiographs are in the DICOM X-ray format. To obtain single limp joints, we separate left and right knee joints (44,558 examples available for training and evaluating predictive models). To increase the number of examples for training purposes only, we used standard data augmentation methods such as a horizontal right-left flip, vertical shift of a maximum of 20% of image height, and brightness rescale of range [0.9, 1.1] of original pixel intensity values.

### Localization model

An accurate object localization within images enables CNN feature extraction to the region of interest (ROI), and that helps minimize extracting irrelevant features and improve computational efficiency. The bilateral PA X-ray images in OAI include all bone structures around a specific joint. For example, each bilateral PA fixed flexion knee radiograph contains almost all femur bone, left kneecap area, right kneecap area, and tibia bone. To assess each kneecap separately, we need to process/classify each knee separately. At the same time, we need to account for variances resulting from using different imaging devices made by different vendors. Current methods used for joint localization in medical images do not address variances like scale and aspect ratio. To address Current methods limitation, we adopted a three-step procedure.

1. A vertical split of raw X-Ray images to isolate left and right Knees in the bilateral PA fixed flexion radiographs (single limp radiographs).
2. Utilize SSD with a pre-trained Mobilenet v1^35^ model for kneecap detection.
3. Resize all cropped images based on the center point and the minimum between width and height of localized joint to a new size of 224 × 224 to unify the scale and to preserve the aspect ratio.

**Figure 6:**
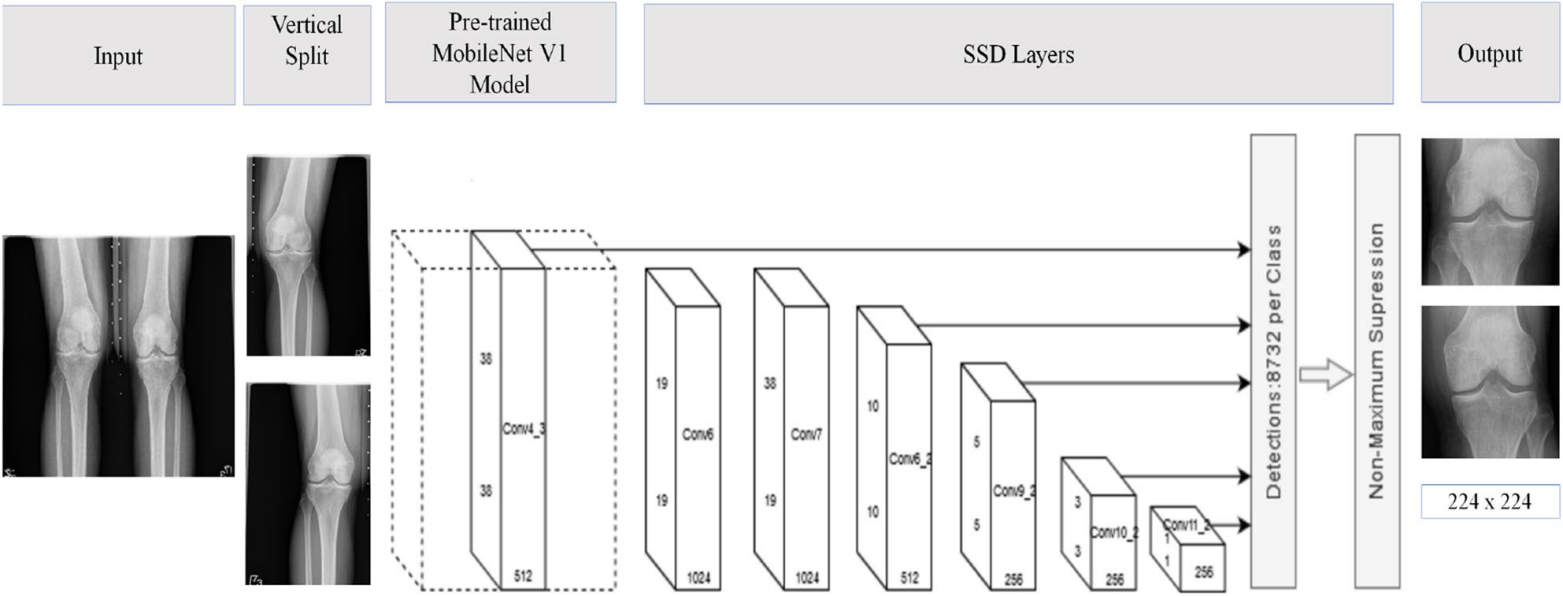
The network architecture for knee localization network.

In general, SSD outperforms methods such as Faster-RCNN^36^ and Yolo^37^ in terms of localization accuracy [mean average precision (mAP)] and real-time localization [frame per second (FPS)]. The SSD architecture is relatively uncomplicated and simpler to train compared to methods that rely on region proposal networks such as Faster R-CNN. The localization architecture is presented in (Fig. 6).

To reduce the number of needed annotated images, we utilized a pre-trained quantized COCO SSD MobileNet v1 model and used the annotated knee joints to fine-tune the MobileNet v1 in the SSD model. MobileNet v1 is a lightweight convolutional neural network architecture from Google known for its relatively small number of parameters (4.2 Million parameters) and low complexity. The low number of parameters enables low processing machines (mobile and embedded vision applications) to use MobileNet v1 in different computer vision tasks.

### Parameter optimized CNN base models

CNN represents a compelling candidate for base model selection in ensemble learning methods, powered by CNN's unparalleled performance in various computer vision tasks such as image classification, object localization, visual question answering (VQA), semantic segmentation, and image captioning. We designed six diverse CNN architectures as a base model with trainable parameters that range between 2,384,549 and between 23,610,661. The Models were trained to predict the KOA severity level (ground-truth) provided by the BU X-ray reading center for OAI 0.2.3 dataset for the variable V00XRKL [BU reading for KL (Grades 0–4)]. In Table 6, we detailed the network architecture for all base models.

**Table 6:** Network architecture of the base models in the stacked ensemble CNN.

Base Model 1				Base Model 2				Base Model 3			
Layer	Filters	Kernel size	Stride	Layer	Filters	Kernel size	Stride	Layer	Filters	Kernel size	Stride
Conv1	32	11 × 11	1	Conv1	32	9 × 9	1	Conv1	32	7 × 7	1
MaxPool1	–	2 × 2	2	MaxPool1	–	2 × 2	2	MaxPool1	–	2 × 2	2
Conv2	64	9 × 9	1	Conv2	64	7 × 7	1	Conv2	64	7 × 7	1
MaxPool2	–	2 × 2	2	MaxPool2	–	2 × 2	2	MaxPool2	–	2 × 2	2
Conv3	96	5 × 5	1	Conv3	96	5 × 5	1	Conv3	96	5 × 5	1
MaxPool3	–	2 × 2	2	MaxPool3	–	2 × 2	2	MaxPool3	–	2 × 2	2
Conv4	128	5 × 5	1	Conv4	128	5 × 5	1	Conv4	128	5 × 5	1
MaxPool4	–	2 × 2	2	MaxPool4	–	2 × 2	2	MaxPool4	–	2 × 2	2
Conv5	256	3 × 3	1	Conv5	256	3 × 3	1	Conv5	256	3 × 3	1
MaxPool5	–	2 × 2	2	MaxPool5	–	2 × 2	2	MaxPool5	–	2 × 2	2
Conv6	256	3 × 3	1	Conv6	256	3 × 3	1	Conv6	256	3 × 3	1
MaxPool6	–	2 × 2	2	MaxPool6	–	2 × 2	2	MaxPool6	–	2 × 2	2
Conv7	512	3 × 3	1	Conv7	512	3 × 3	1	Conv7	512	3 × 3	1
MaxPool7	–	2 × 2	2	MaxPool7	–	2 × 2	2	MaxPool7	–	2 × 2	2
Flatten				Flatten				Flatten			
Fc1	2048			Fc1	2048			Fc1	1024		
Dropout	0.1			Dropout	0.1			Dropout	0.1		
SoftMax (Number-of-Class = 5)											

Base Model 4				Base Model 5				Base Model 6			
Layer	Filters	Kernel size	Stride	Layer	Filters	Kernel size	Stride	Layer	Filters	Kernel size	Stride
Conv1	32	3 × 3	1	Conv1	32	11 × 11	1	Conv1	32	5 × 5	1
Conv2	32	3 × 3	1	MaxPool1	–	2 × 2	2	Conv2	32	5 × 5	1
MaxPool1	–	2 × 2	2	Conv2	64	9 × 9	1	MaxPool1	–	2 × 2	2
Conv3	64	3 × 3	1	MaxPool2	–	2 × 2	2	Conv3	64	3 × 3	1
Conv4	64	3 × 3	1	Conv3	96	7 × 7	1	Conv4	64	3 × 3	1
MaxPool2	–	2 × 2	2	MaxPool3	–	2 × 2	2	MaxPool2	–	2 × 2	2
Conv5	96	3 × 3	1	Conv3	128	5 × 5	1	Conv5	96	3 × 3	1
Conv6	96	3 × 3	1	MaxPool4	–	2 × 2	2	Conv6	96	3 × 3	1
MaxPool3	–	2 × 2	2	Conv4	256	3 × 3	1	MaxPool3	–	2 × 2	2
Conv7	128	3 × 3	1	MaxPool5	–	2 × 2	2	Conv7	128	3 × 3	1
Conv8	128	3 × 3	1	Conv5	256	3 × 3	1	Conv8	128	3 × 3	1
MaxPool4	–	2 × 2	2	MaxPool6	–	2 × 2	2	MaxPool4	–	2 × 2	2
Conv9	256	3 × 3	1	Conv6	512	3 × 3	1	Conv9	256	3 × 3	1
Conv10	256	3 × 3	1	MaxPool7	–	2 × 2	2	Conv10	256	3 × 3	1
MaxPool5	–	2 × 2	2					MaxPool5	–	2 × 2	2
Conv11	256	3 × 3	1					Conv11	256	3 × 3	1
Conv12	256	3 × 3	1					Conv12	256	3 × 3	1
MaxPool6	–	2 × 2	2					MaxPool6	–	2 × 2	2
Flatten				Flatten				Flatten			
Fc1	1024			Fc1	1024			Fc1	1024		
Dropout	0.1			Dropout	0.1			Dropout	0.1		
SoftMax (number-of-class = 5)											

### Introducing diversity into base models

To introduce diversity among different CNN classifiers, we varied Conv2D layers kernel size, training batch, and hyperparameters values. To ensure maximum performance for each base model, we used Bayesian optimization to optimize all different architectures' hyperparameters. The hyperparameters included the *training batch size, kernel size, stride size, activation function type, dropout rate, l2 regularization penalty, number of dense layers, and number of nodes in each dense layer.* To reduce overfitting, we used a single dropout layer in every model. To account for the skewed distribution of KL levels, we used a heuristic inspired by logistic regression in rare events data^38^. We trained base models to minimize categorical cross-entropy loss function using the Adam optimizer with a criterion-based reducing learning rate starting from 0.001.

### Ensemble model

Ensemble learners improve generalization by combining several weak learners to account for noise, bias, and variance, techniques such as bagging, boosting, unweighted average, majority vote, and stacking. Bagging (Bootstrap Aggregation) is a two-step operation. In the first weak learners, classify samples drawn with replacement from the training data. In the second, the ensemble technique aggregates the output of the base models. In general, bagging (e.g., Random Forest) improves accuracy, reduces variance, and increases stability. Boosting converts weak learners into stronger learners using different metaheuristics. In general, boosting aims at reducing bias and variance (e.g., AdaBoost, Gradient Boosting).

**Figure 7:**
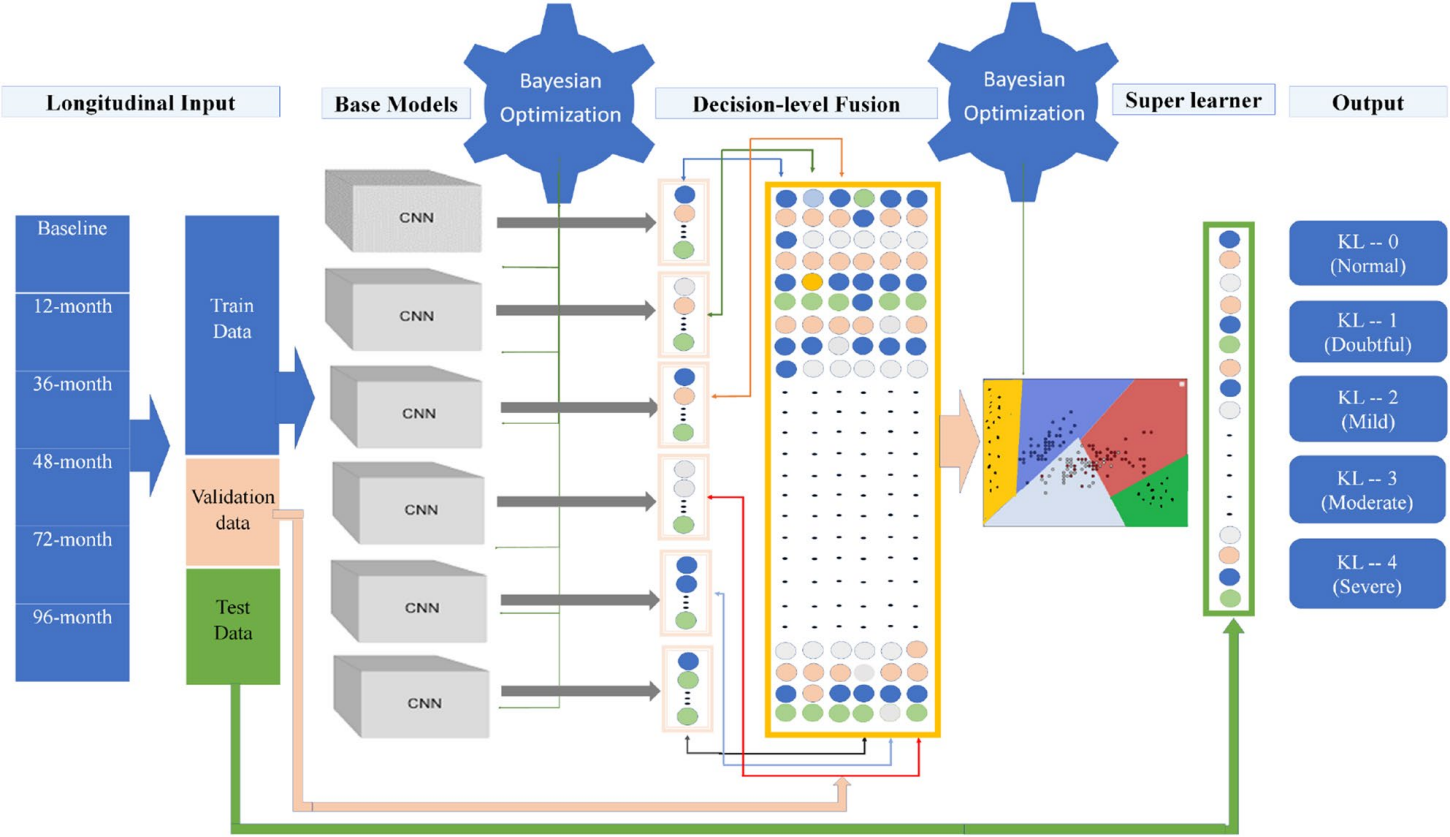
The network architecture for classification network.

The unweighted average combines predictions at the SoftMax layer level with equal weights. The majority vote combines predictions at the SoftMax layer's output with equal weights (prediction vote count). Stacking or stacked generalization combines similar or different classification or regression base models using a meta-learner. In general, stacking allows mixing different types of models at different levels (base or meta-learner level) to boost accuracy (Fig. 7). The super learner is a particular case of stacking where the super learner is used to find the base learners' optimal weights in the ensemble model by minimizing the loss function based on the cross-validated output of the base learners^39^.

To select base models for the super learner ensemble model, we used Eigen-CAM to check for models that show consistent learned patterns. We kept models that show learned patterns for diversity check and excluded models that show no learned or consistent visual patterns. Moreover, we excluded all models that do not add to the overall diversity of the selected models. For the super learner choice, we experimented with classical machine classifiers such as support vector machines (SVM)^31^, Random Forest (RF)^32^, and Gradient Boosting Machines (GBM)^33^.

### Prediction explanations

As the case with most DL methods, CNN's inner workings are usually referred to as a black-box operation. CNN models end users raise justified concerns such as generalization capability and the ability to decode prediction failure. We utilize a state-of-the-art approach Eigen-CAM to provide class activation maps for CNN output decisions (class discriminative) to address such concerns. Eigen-CAM generates class activation maps (CAM) for CNN prediction using the learned feature's principal components at the last Conv2D layer in CNN models.

Eigen-CAM is an intuitive class-independent tool that can work with any CNN architecture without the need to modify or retrain the model. The class independence property enables Eigen-CAM to visualize learned patterns independent from dense layers and the SoftMax layer (does not require a correct decision at the CNN model's output). This property enables decoding prediction failure and helps with the design process of CNN architectures. In general, Eigen-CAM provides a better and consistent explanation with less computation (does not require any backpropagation operations to generate visual explanations).
